# Dental Health Utilization in Palau: Feasibility of an Oral Cancer Screening Program

**DOI:** 10.5334/aogh.4174

**Published:** 2023-09-20

**Authors:** Katherine Rieth, Angela Sy, Scott McIntosh, Edolem Ikerdu, AnaPaula Cupertino, Timothy D. Dye, Camille Anne Martina

**Affiliations:** 1 Department of Otolaryngology, University of Rochester Medical Center, Rochester, NY, USA; 2 John A. Burns School of Medicine, University of Hawaii at Manoa, Honolulu, HI, USA; 3 Department of Public Health Sciences, University of Rochester Medical Center, Rochester, NY, USA; 4 Division of Primary & Preventive Health, Palau Bureau of Public Health, Koror, PW; 5 University of Rochester Medical Center, Rochester, NY, USA; 6 School of Medicine and Dentistry, University of Rochester Medical Center, Rochester, NY, USA

**Keywords:** oral cancer, cancer screening, Palau, dental health, betel nut

## Abstract

**Background:**

Cancer is the second leading cause of death in the Western Pacific region. The prevalent tradition of chewing betel nut in Palau, an island nation in this region, is a risk factor in the development of oral cancer. Oral cancer is the fifth most common cancer in Palau, and the prognosis can be improved with early detection facilitated by visual inspection of the oral cavity by dentists. The purpose of this study is to assess the feasibility of oral cancer screening using existing dental health infrastructure in Palau.

**Methods:**

A mixed methods approach was used to explore topics related to the use of dental care resources in Palau. Primary outcome measures were collected using an electronic survey with closed- and open-ended questions addressing dental health utilization as well as barriers and facilitators to accessing dental care. Secondary measures assessed knowledge, attitudes, and beliefs about betel nut use and oral cancer. Open-ended survey questions were analyzed and coded to develop themes based in grounded theory.

**Results:**

Two hundred twenty-three surveys were completed. The mean age was 42.7 years, 80% identified as female, and most (94.3%) report having seen a dentist in Palau. Dental care is seen as important (mean score 82.3/100), and 57.9% reported it was easy to access a dentist. Themes regarding facilitators include multilevel resources and transportation. Themes regarding barriers include cost and availability of dentists/appointments. Approximately half of the respondents were current users of betel nut.

**Conclusion:**

Our results suggest facilitators are in place to promote seeking and obtaining dental care; however, existing infrastructure may not support an oral cancer screening program. These data provide important areas to address that can improve access and support the implementation of oral cancer screening through existing dental care in the future.

## Introduction

**Figure 1:**
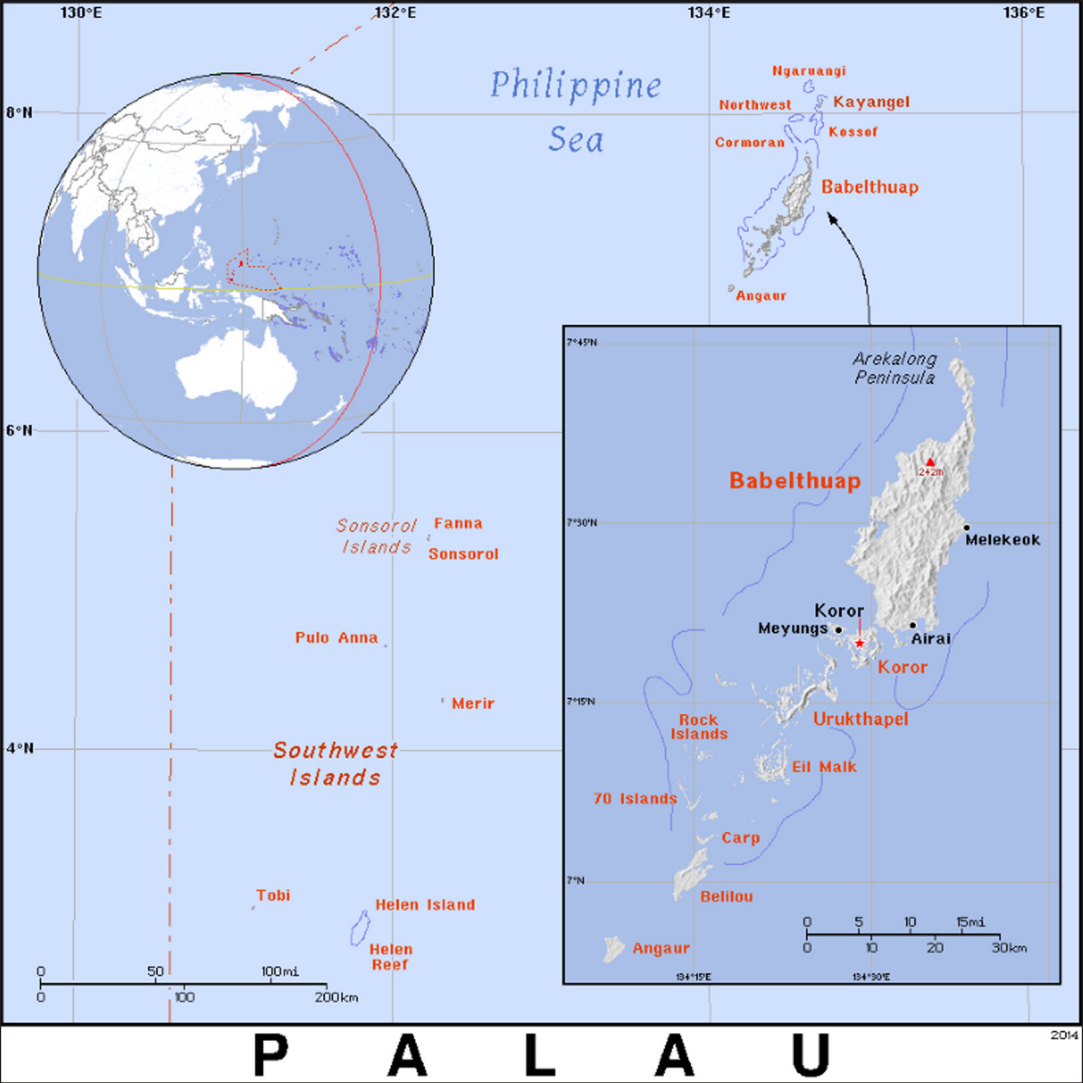
Map of Palau.

Oral cancer is the fifth most common cancer in the Republic of Palau (Figure 1), an island nation in the United States–Affiliated Pacific Islands (USAPI), with poor prognosis that can be improved with early detection [1,2,3]. Cancer is the second leading cause of death in USAPI due to multiple barriers across the cancer control continuum, including detection, which leads to late-stage presentation and low likelihood of survival [4,5]. Challenges for Palauans in early cancer detection include remote populations requiring unpredictable land and sea travel as well as limited human and technical resources. Due to the relative geographic isolation of Palau from major medical centers, efforts to improve early cancer detection for this population are of particular importance.

Risk factors for developing oral cancer include: (1) both smoked and smokeless tobacco, (2) the excessive consumption of alcohol, and (3) chewing the seed of the *areca catechu palm*, commonly referred to as betel nut [6]. Chewing betel nut is an important cultural identifier and integral part of many cultural rituals in Palau [7,8]. There is a high prevalence of betel nut use reported among Palauans (68.5%–76%) [9,10], and 88% of betel nut users also add tobacco to their quid [5,9,10], which compounds the carcinogenic effect of betel nut [9,10]. The associations between betel nut chewing and oral potentially malignant disorders (OPMD), including oral cancer, have been noted in other areas of USAPI, including Guam and the Commonwealth of the Northern Mariana Islands (CNMI) [10].

Early detection of oral cancer is essential and can be facilitated by visual inspection of the oral cavity by dentists or primary care physicians. The National Cancer Institute (NCI) finds that, overall, late stages of oral cancers (stage III/IV) have a 5-year survival rate as low as 9.5% [1,11]. Median overall survival is reported as 30 months in patients born in Pacific Island territories compared to 54 months for the entire population in this study [12]. In one study based in CNMI [12], oral cavity cancers contributed to 13.3% of cancer deaths compared to 1.0% in the United States. Of those oral cancer patients in this study, 78.6% presented with advanced disease (stage III/IV), suggesting there are disparities and unequal access to early detection services, such as dental care.

On a global level, inequalities in dental service utilization and oral/dental health [13,14] are driven by cultural, economic, social, and environmental determinants of health, especially in developing countries [15]. Previous research to assess disparities in the utilization of dental services has shown that enabling factors are key predictors of utilization. Significant socioeconomic predictors include: (1) education [16,17,18,19,20], (2) dental health insurance coverage [16,17,21,22], and (3) social support [22,23,24]. As such, the existing literature supports the need for improved oral cancer screening programs in Palau [4,10,25,26]. Efforts to assess and improve outcomes of oral cancer in adjacent regions of the Pacific [26] have utilized questionnaires to obtain demographic data and betel nut chewing behaviors as well as offering onsite oral examinations. Our study aims to address gaps in knowledge about how adults in Palau use dental services, including enabling factors, challenges, and barriers. These data, in the context of how this community understands oral cancer, can then be used to assess the feasibility of developing a screening protocol for oral cancer utilizing current dental health providers and infrastructure.

## Methods

### Study design

The original study design was an in-depth in-person qualitative examination about oral cancer and quantitative surveys. Due to limitations in engaging in participatory research internationally during the COVID-19 pandemic coinciding with this study (Spring 2020–Summer 2021), the original research methodology of a mixed methods convergent study was significantly modified to reach participants remotely through the Internet. We chose Facebook as it had the potential to target certain demographic characteristics through Facebook ads to facilitate research recruitment efforts [27]. Through our history of collaboration, social media is widely used and provided an ideal opportunity to recruit participants for a remote survey study.

The original key informant and focus group interview guides were modified and incorporated into an Internet-based open-ended written questionnaire and multiple-choice survey. The questions were formulated using Andersen’s behavioral model of health service use as a framework, which provides multilevel factors that facilitate or impede an individual’s access to healthcare services. A systematic review on dental services use based on the Andersen model found that predisposing characteristics (e.g., gender, age, marital status or ethnicity) and need factors were linked to dental utilization [28]. In this study, Andersen’s model will be used to explore and predict a sample of Palauans’ intentions and behaviors as they utilize healthcare services.

### Recruitment

**Figure 2:**
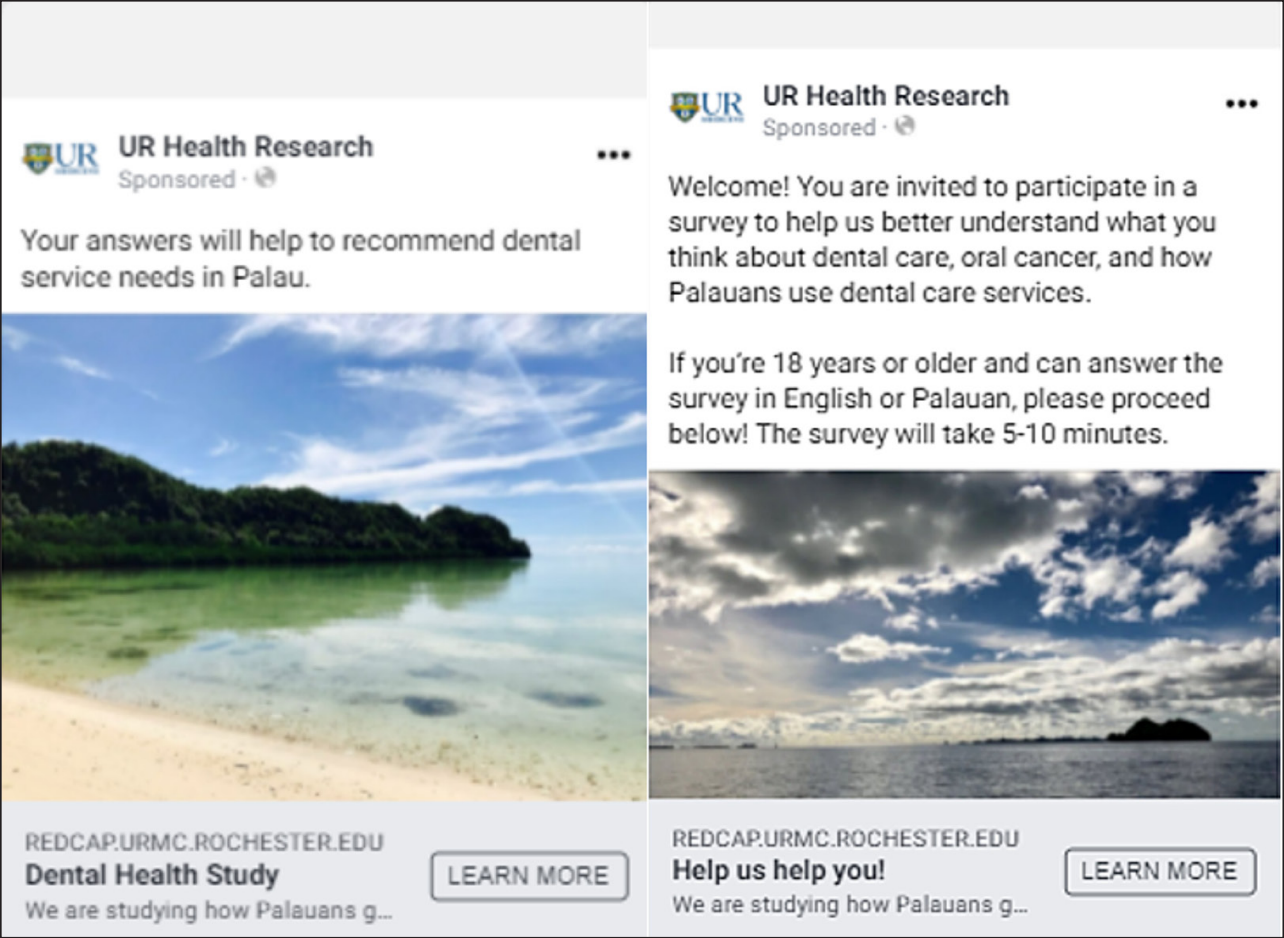
Examples of Facebook ads used for recruitment.

Facebook users aged 18 and older within Palau were invited to complete the survey online through a secure site. Facebook ad invitations (Figure 2a and 2b) were shareable, allowing social media users to re-post the invitation in the form of snowball sampling. The survey and consent forms were made available in both English and Palauan. All questions had the option of text-to-speech functionality to mitigate limitations of literacy. The survey was available through Facebook ads from May 23, 2021, through May 31, 2021, for a total of nine days. The survey period was limited solely by funding availability. There was no financial compensation provided to participants.

Data were collected through a secure data collection site hosted by the University of Rochester using the REDCap system (Research Electronic Data Capture, Vanderbilt University). Informed consent to participate in the survey was required and obtained electronically, and the study was considered minimal risk. This study was approved and determined to be exempt from review by the Palau Institutional Review Board and University of Rochester Research Subjects Review Board (approved May 20, 2021, STUDY6206).

### Measures

The survey was comprised of closed- and open-ended questions that investigated current use of dental services (access, frequency, and purpose) and attitudes and beliefs about betel nut use and oral cancer. Closed-ended questions included Likert scales, sliding scales, and multiple-choice responses. Demographic information was also collected, including education level, age, gender, and island of residence within Palau.

### Data analysis

The open-ended survey responses were analyzed and coded independently by two members of the research team (KR, AS) using open coding processes and inductive reasoning based in grounded theory to discover emerging and important themes. Open coding of the free-text responses involved (a) each of the two coders independently identifying themes of the responses, (b) discussing and agreeing on initial themes, (c) grouping similar thematic codes into summarizing categories, (d) identifying conceptual codes, and (e) final agreement on all levels of thematic codes by the coders. Both researchers coded the free-text responses independently. Then both coders reviewed their codes, and discrepancies were resolved through multiple discussions to reach consensus. A finalized codebook with code frequencies was created to identify and describe the final emergent themes on feasibility of dental services in Palau.

Statistical analyses were conducted using SPSS (IBM SPSS Statistics, version 9.8.0.0). The primary outcome was defined as ease of seeking dental care. Responses of “neutral,” “somewhat easy,” and “very easy” were considered positive. Demographic characteristics were evaluated as predictors. These included age, gender, level of education, and location of residence. Each predictor was evaluated in cross tabulation individually as well as through logistic regression.

## Results

Reach is defined as the number of people who saw the advertisement. This was calculated as 40,016, which represents approximately 200% penetration of the estimated available Facebook users in Palau. The response rate, as calculated by completed surveys to link clicks, was 10.4%. The cost per link click was US$0.28, making the cost per completed survey approximately US$2.90.

At the conclusion of the study period, 229 participants had completed the study. Of these, five were excluded due to being incomplete, and three more were excluded as duplicates for a total of 223 unique completed survey responses. The age range of respondents was 18–75 years, with a mean age of 42.7 years. One hundred seventy-seven (80.1%) respondents identified as female. The majority (60.4%) resided on Koror, the most populous state in Palau. Sixty percent of respondents had some university/college level education (attended and/or graduated). The vast majority (94.3%) report having seen a dentist in Palau (Table 1). When asked how important regular dental care, such as cleanings and check-ups, is to them as individuals, respondents were given a sliding bar scale from 1 to 100, with 100 operationalized as “very important.” Overall, dental care was seen to be important to respondents (mean score 82.27; median score 96.0). Correspondingly, most respondents reported having seen a dentist in Palau at least three times or more (*n* = 185; 83.7%) in their lifetime. Most respondents felt that it was easy to see a dentist, with 131 respondents rating it “very easy” or “somewhat easy” (59.2%). Including those who answered “neutral,” 74.2% of respondents felt there were no major barriers to seeing a dentist in Palau (*n* = 164).

**Table 1:** Characteristics of participants.


CHARACTERISTICS		*N*	%

Age	18–34	63	28.3%

	35–44	58	26.0%

	45–54	58	26.0%

	55–64	33	14.8%

	65–74	10	4.5%

	75 and older	1	0.4%

	Mean 42 years		

	Range 18–75		

	Total	223	100%

Gender	Male	44	19.9%

	Female	177	80.1%

	Total	221	100%

Level of education	Did not complete high school	7	3.2%

	Completed high school	37	16.9%

	Attended university but did not complete	66	30.1%

	Graduated from university	79	36.1%

	Degree beyond university	30	13.7%

State of residence	Koror	135	60.5%

	Other state	88	39.5%

**Total**		**223**	**100%**


Predictors of seeking dental care are described in Table 2. Despite the lack of statistical significance, these variables were then evaluated in a logistic regression, which also demonstrated no significant predictors in a positive “yes” response to ease of seeking dental care. Regarding betel nut use, 54.6% (*n* = 121) identified as current users of betel nut. Most respondents reported that betel nut was connected to oral cancer (*n* = 142, 62.3%), and only 16.3% (*n* = 36) believed that betel nut was not a cause of oral cancer. Over half of the respondents (*n* = 134, 60.6%) reported having known someone with oral cancer. The majority reported that they would seek care from a doctor or dentist (*n* = 213, 96.4%) if they thought they had oral cancer. Likewise, most respondents indicated their preferred location for an oral/dental examination would be a dentist’s office or community health center (*n* = 212, 95.9%), compared to an in-home examination (*n* = 2, 0.9%) or a community center (*n* = 3, 1.3%). Only 24.5% (*n* = 55) would allow someone to come to their home to examine their mouth and teeth. The most identified causes of oral cancer by respondents are smoking tobacco, chewing tobacco, genetics, and betel nut. Logistic regression did not identify any significant predictors of betel nut use among education level, age group, state of residence within Palau, or belief that betel nut causes oral cancer.

**Table 2:** Predictors of perceptions of access to dental health services.


CATEGORY	VALUE	*N* (%)	*P* VALUE

Seeking dental care is easy	No	92 (42%)	

	Yes	**127 (58%)**	

Education category	High school or less	73 (33.3%)	0.446

	At least some university education	**146 (66.7%)**	

State of residence	Koror	**130 (59.4%)**	0.186

	Other state	89 (40.6%)	

Age group	18–34	63 (28.5%)	0.332

	35–44	57 (25.8%)	

	45–54	58 (26.2%)	

	55–64	32 (14.5%)	

	65 and older	11 (5.0%)	


### Free response results

#### Barriers

The thematic analysis of open-ended responses yielded 32 codes under barriers to dental care. The five most common codes by frequency addressed issues of access and obligations. Four of the five most common codes were identified as access concerns. Overlap between difficulty scheduling appointments and work obligations further complicated an individual’s ability to schedule an appointment. During the coding process, the decision was made to distinguish specific mention of work and employment from general and other scheduling difficulties. Because this distinction of work-related barriers resulted in the fourth most frequent code, it is likely significant. Based on these codes and code frequencies, four themes were identified pertaining to barriers and facilitators to dental care utilization.

### Theme 1: Lack of available appointments and number of dentists

The difficulty in making prompt appointments was a pervasive theme among respondents when asked about barriers to seeking dental care in Palau. The wait time to see a dentist, that is, the time between calling to make the appointment and when that appointment could be scheduled, was felt to be quite long. This perception seemed to apply to both preventive and treatment-oriented visit requests.

*Six to 8 months waiting for your appointment, to see a doctor’s or dentist*.

*I have to make an appointment and I have to wait for a very long time for them to contact me so by the time they call I have to cancel because something comes up*.

*Dental visitation list is very long that it takes about 4 to 5 months for anyone to get their teeth cleaned. Sometimes you just forget about your appointment because you waited so long*.

In addition to long wait times until an appointment could be made, many respondents noted the hours that dentistry clinics were open were a limiting factor. Obligations such as employment/work and childcare were identified as barriers to accessing dentists during these hours.

*I do wish the dental would be open on weekends to serve or help those people with [a] tight working schedule like me*.

*Work schedule and that the dental is closed on weekends*.

Many respondents reported that the difficulty in making prompt appointments was due to a lack of dentists available in Palau. This deficit was frequently identified as part of the difficulty in making appointments and a barrier to care.

*If we could have more dentists*.

*More dentists, more supplies, more resources*.

*Availability of dentists (need more)*.

*Scheduling, fees, lack of supplies and/or shortage of dentist*.

### Theme 2: Seeking dental care is limited by cost

The costs of obtaining dental care were another frequently cited barrier to access among the respondents. Unfortunately, the format of the survey did not encourage detailed commentary on responses. Therefore, many respondents simply wrote “cost” or “financial” for this question. The mention of cost was the third most frequent code from the category of barriers to care.

*It’s always the cost and whether my medical savings account with the gov’t can cover it*.

*Dental service in Palau is very expensive*.

*Too expensive and always fully booked*.

#### Facilitators

Nineteen codes were developed under the category of facilitators. The three most frequent codes addressed available resources that facilitated seeking and obtaining dental care as well as available services that were perceived to promote an individual’s ability to be seen by a dentist. The resource-related codes identified available transportation and insurance programs as supporting access to dental care. The service-related code addresses systems put in place by dental clinics, such as call reminders, walk-in availability, and a cancellation list, that facilitate dental care. One of the four most frequently identified codes was “none,” which indicated that the respondent did not leave this blank but identified no known facilitators to care. This response was felt to be thematically different from a non-response (i.e., left blank).

### Theme 3: Resources at multiple levels exist to facilitate obtaining dental care

Respondents identified a number of resources that promote the ability to get dental care. These resources exist at multiple levels as described in the socioecological model. For example, at the government/policy level, there are insurance programs to help with the cost. At an individual level, having transportation was seen as a facilitator to care.

*Local health insurance so it won’t come directly out of my pocket*.

*Sometimes have enough government medical savings to cover cost*.

*I have my own vehicle to drive to the dentist*.

*I have transportation that I can use to go to the dentist if I need to*.

### Theme 4: Dental clinics provide services that promote obtaining dental care

Dental clinics have systems in place to help facilitate dental care. Respondents identified system elements in the dental care infrastructure, such as reminder calls and availability for emergency appointments, as ways that dental clinics help promote receiving care.

*It is just a phone away; all I need is to call to make an appointment*.

*I can call anytime for appointments or walk in for any emergencies*.

*You can easily make an appointment. They will call you to remind you for your appointment*.

*Dental registrar calls me as soon as there is a cancellation*.

**Table 3:** Themes addressing facilitators and barriers to dental health utilization.


	KEY THEMES	DESCRIPTION	DEMONSTRATIVE QUOTES

***Barriers***	Lack of available appointments and number of dentists.	The long wait time for an available appointment to see a dentist and the hours of availability of dental clinics. The lack of dentists available creates long wait.	“I have to make an appointment and I have to wait for a very long time for them to contact me so by the time they call I have to cancel because something comes up.”

	Seeking dental care is limited by cost.	Financial constraints and the cost of dental care are prohibitive.	“Too expensive and always fully booked.”

***Facilitators***	Resources at multiple levels exist to facilitate obtaining dental care.	Private and public medical/dental insurance programs help offset the cost of dental care. Having personal and/or available transportation.	“Sometimes have enough Government medical savings to cover cost.” “I have my own vehicle to drive to the dentist.”

	Dental clinics provide services that promote obtaining dental care.	Phone call reminders, cancellation lists, and walk-in for emergencies promote obtaining care.	“I can call anytime for appointments or walk in for any emergencies.”


## Discussion

This study found that while there are some mechanisms and resources that facilitate seeking dental care, there are also perceived barriers to dental care access that are considerable for adults in Palau. Most (95%) respondents indicated they would prefer to be seen by a dentist or physician in a clinical setting for cancer concerns. The importance of regular dental care, such as cleanings, was also rated high among respondents. Additionally, most (94.3%) respondents reported having seen a dentist in their lifetime, and the majority (70.5%) felt there were no major barriers to seeing a dentist. There were no significant predictors for what made seeking dental care seem easy [13]. The results suggest that those seeking dental care are able to do so.

Thematic code frequency addressed facilitators to dental care that could be categorized as enabling resources according to the Andersen model. One of the three most frequent codes among facilitators was the existence of national and other insurance programs (*N* = 24). Many respondents cited Medical Savings Accounts (MSA) and National Health Insurance (government-sponsored) programs that helped offset the out-of-pocket costs for dental care. Access to government-sponsored medical/dental insurance coverage was also reported as a facilitator to dental care in other studies of dental service utilization in various countries, including low- and middle-income countries (LMICs) [13,14,29]. This information provides a possible strategy for Palau’s Ministry of Health to adopt in outreach and education to the public, emphasizing existing programs that are available to assist with the costs of dental care.

Open-ended responses that centered on barriers to access draw attention to not being able to make a timely dental appointment (*N* = 107) and highlight the role of “need factors,” per the Andersen model, in seeking dental care [28]. Many respondents felt that the extended time between calling for an appointment and securing an appointment was prohibitively long. This may deter an individual from seeking care at all, and some noted that their initial oral health concerns at the time of making the appointment might worsen during the wait time. A systematic review of dental care seeking behavior unexpectedly found that need factors such as oral health, oral pain, decayed teeth, or need of treatment were not consistently associated with dental service use [28]. In our study, need factors appear to be mitigated by perceptions of enabling resources, such as availability of dental services. Overall, the difficulty accessing dental care due to lack of available appointments was often attributed by the respondents to the lack of available dentists in Palau.

However, the lack of dentists in Palau is not the problem. In 2020, the population of the Republic of Palau was 18,092 (datacatalog.worldbank.org, accessed July 20, 2022) with the number of active dentists estimated at seven. According to the World Health Organization, the ideal ratio of dentists in a population is 1:7,500 [30]. Given the number of dentists reported to be working in Palau and the most recent population estimate, this ratio is met (1:2,586). For comparison, the ratio of dentists in the United States is 1:1,650 [31]. Furthermore, most dentists in Palau have walk-in availability for emergencies, which was cited by some as a facilitator to care. However, many respondents experience a prolonged time between calling to schedule an appointment and the scheduled appointment, leading to the perception of too few dentists. This perceived limited access to dentists resulted in patients who would often forget appointments or develop progressive problems with their teeth.

The open-ended responses elaborate on accessibility to dental care and challenge the interpretation of the quantitative results. These responses suggest that important barriers to regular dental care would affect the feasibility of an oral cancer screening program that relies solely on the existing dental care infrastructure. When interpreting these results, it is important to remember that these are perceptions of limited access that require further exploration and clarification. These issues are especially important in the context of oral cancer screening using existing dental care infrastructure, as suspicious oral lesions can worsen and/or transform into more worrisome entities over the wait time of two to eight months cited by many respondents.

### Limitations

Facebook was the only recruitment method utilized in this study, so responses came from a convenience sample. In addition, the duration of the survey was limited exclusively by availability of funding. Results cannot be generalized to all adult Palauans due to the small sample size and are limited to those who have access to the Internet and social media. Demographic groups that were overrepresented were females, higher education levels, and those 18 to 54 years old. Although Topolovec-Vranic and colleagues [32] did not find a difference between male and female recruitment in their scoping review of social media recruitment in medical research studies, our sample does support female selection bias in our sample characteristics. Predisposing characteristics to seeking dental care, as defined by the Andersen model, found in other studies of dental utilization in LMICs include adults who identify as female and have higher education levels [13,14]. Although these characteristics were highly represented in our sample, our analysis did not find these to be significant predisposing characteristics to seeking dental care. Males and the elderly are underrepresented in this sample, which may reflect differences in interest in the topic as well as social media patterns of use. Respondents’ geographic distribution reflected the general population, where the majority of respondents (60.5%) were from the urban center of Koror (compared to 70% of Palau’s general population).

The online survey included open-ended questions, which may limit the trustworthiness of the qualitative research; free-text responses within structured surveys rarely produce data rich enough to achieve the characteristics of rigorous qualitative research [33]. Previous studies support the use of open-ended questions as an adjunct in survey research for reasons that also apply to our studies [34,35]. The open-ended responses provide deeper insight into explaining and contextualizing the close-ended survey responses, as well as providing direction for future exploration. The information from the open-ended responses provides an initial perspective, from the standpoint of the community [36], in lieu of in-person engagement and query, of barriers and facilitators to dental care that should be further enriched with follow-up in-depth interviews and/or focus group interviews.

### Future directions

With the relatively high incidence and prevalence of oral cancer in Palau, an oral screening program would be invaluable to reducing the morbidity and mortality from this disease. These findings provide many trajectories for future studies. Toward the goal of developing an oral cancer screening program using existing dental care infrastructure, a cost-benefit analysis for screening through regular dental health exams versus the cost of treatment for oral cancer would be a useful adjunct to this investigation. The availability and reach of existing insurance and MSA programs would also be important. This investigation would also benefit from deeper exploration through qualitative methods, i.e., interviews and/or focus groups, to further clarify the perceived difficulties in securing dental appointments from both patient and dental health provider perspectives. The survey component of this investigation would benefit from a broader and larger population sample in an effort to make the results more generalizable to the Palau population overall.

Recommendations about implementing oral cancer screenings will require exploring in greater depth the decisions to seek care among high-risk individuals, that is, those who chew betel nut and/or tobacco, especially with concomitant use of alcohol. The use of telehealth in oral cancer screening has also been investigated in low-resource communities and would be another approach to investigate for screening in Palau [37,38,39,40,41]. A multilevel approach to screening that implements elements of social networks, social organizations, community infrastructure, and government public policy should also be further investigated in regard to dental utilization in Palau.

## Conclusion

Dental care is important to Palauans, and most would prefer to have this occur in a clinical setting. The themes from this study uncovered enabling resources for dental utilization; however, there are barriers that suggest the existing infrastructure and access to dental care present challenges to initiating an oral cancer screening program. These results provide important areas to address in the delays associated with accessing dental care.

This study provided a theoretically guided framework using Andersen’s Healthcare Utilization Model to examine dental utilization in Palau. To our knowledge, this approach using quantitative and qualitative descriptive results to assess dental utilization on Palau has not been published. This study also uses the Andersen model to add a level of insight about dental services and their use in Palau not obtained in previous studies.
